# Modelling focused electron beam induced deposition beyond Langmuir adsorption

**DOI:** 10.3762/bjnano.8.214

**Published:** 2017-10-13

**Authors:** Dédalo Sanz-Hernández, Amalio Fernández-Pacheco

**Affiliations:** 1 Cavendish Laboratory, University of Cambridge, JJ Thomson Cambridge, CB3 0HE, United Kingdom

**Keywords:** adsorption isotherm theory, BET model, continuum model, focused electron beam induced deposition, 3D nanoprinting, Langmuir model

## Abstract

In this work, the continuum model for focused electron beam induced deposition (FEBID) is generalized to account for multilayer adsorption processes. Two types of adsorption energies, describing both physisorption and spontaneous chemisorption, are included. Steady state solutions under no diffusion are investigated and compared under a wide range of conditions. The different growth regimes observed are fully explained by relative changes in FEBID characteristic frequencies. Additionally, we present a set of FEBID frequency maps where growth rate and surface coverage are plotted as a function of characteristic timescales. From the analysis of Langmuir, as well as homogeneous and heterogeneous multilayer maps, we infer that three types of growth regimes are possible for FEBID under no diffusion, resulting into four types of adsorption isotherms. We propose the use of these maps as a powerful tool for the analysis of FEBID processes.

## Introduction

Focused electron beam induced deposition (FEBID) is a direct-write nanolithography technique, based on the local decomposition of gas molecules adsorbed on a substrate and induced by the interaction with a focused beam of electrons [1–3]. FEBID does not require masks or templates, it can achieve sub-10 nm spatial resolution [4–5], and it has the unique ability to fabricate complex three-dimensional nanostructures [6–9].

Recent key progress on FEBID includes the growth of pure metallic nanostructures by mixing precursor and reactive gases [10–14] and exploiting autocatalytic effects [15–16], the design of improved synthetic precursor molecules [17], the usage of new gas injector systems [18], the synthesis of compounds [19–20], and the application of FEBID systems in several areas of nanotechnology [8,21–23], just to cite a few. Moreover, significant effort is now dedicated to enhance the predictability of FEBID processes by means of modelling, which means a shift from a trial-and-error approach, to a quantitative, model-guided 3D nanoprinting method. This progress includes the development of a Monte Carlo model to simulate gas flow surface distribution when delivered from an injector [24], code that analytically and numerically solves FEBID continuum models [25], a hybrid Monte Carlo-continuum model to predict and guide the growth of 3D nanostructures [26], and a molecular dynamics model to give an account of FEBID at the molecular level [27]. A key ingredient to much of this recent progress is the FEBID continuum model [25], which describes the time evolution of adsorbate concentration on a substrate as a function of the various processes comprising FEBID. This model describes nanostructure geometry and growth rates as a function of experimental parameters, such as current and gas flux, helping to explain the underlying growth mechanisms observed in experiments. However, in the present form, the FEBID continuum model cannot account for the different purities in FEBID deposits observed for various growth regimes when considering a single adsorbate, and has been restricted to physisorption processes, except for a few exceptions [28–29]. Relevant effects present in common FEBID precursors, such as autocatalytic effects [15–16,30–32] cannot be described either. Moreover, current continuum models restrict their range of applicability to Langmuir adsorption, where a maximum of one monolayer can be adsorbed [1]. Multilayer adsorption is, however, common in standard vacuum science studies, usually conducted at low temperatures [33–36], in cryogenic FEBID [37] and at higher temperatures for precursors with low volatility [1]. The breakage of the Langmuir model is also common for low adsorbate concentrations on heterogeneous substrates [38].

A better understanding of the FEBID underlying processes is required, including advances in the design of superior molecules for deposition under electron irradiation [39]. This demands for new frameworks which describe FEBID processes more generally and under a wide range of experimental conditions [40]. Here, we generalize the FEBID continuum model, going beyond Langmuir adsorption, that is, allowing the system to form adsorbate coverages above one monolayer. By introducing two types of adsorption energies, we simulate FEBID processes involving both chemi- and physi-adsoption. Under this model, we investigate what conditions are necessary for either mono- or multi-layer adsorption by analytically calculating the stationary state of the system under no diffusion. The findings are explained taking into account the key timescales involved in the process. Finally, we present general maps for average adsorbate concentration and growth rate as a function of fundamental growth parameters, which can be compared with experimental data in order to identify different FEBID regimes.

## Results and Discussion

### Multilayer FEBID model

**Figure 1:**
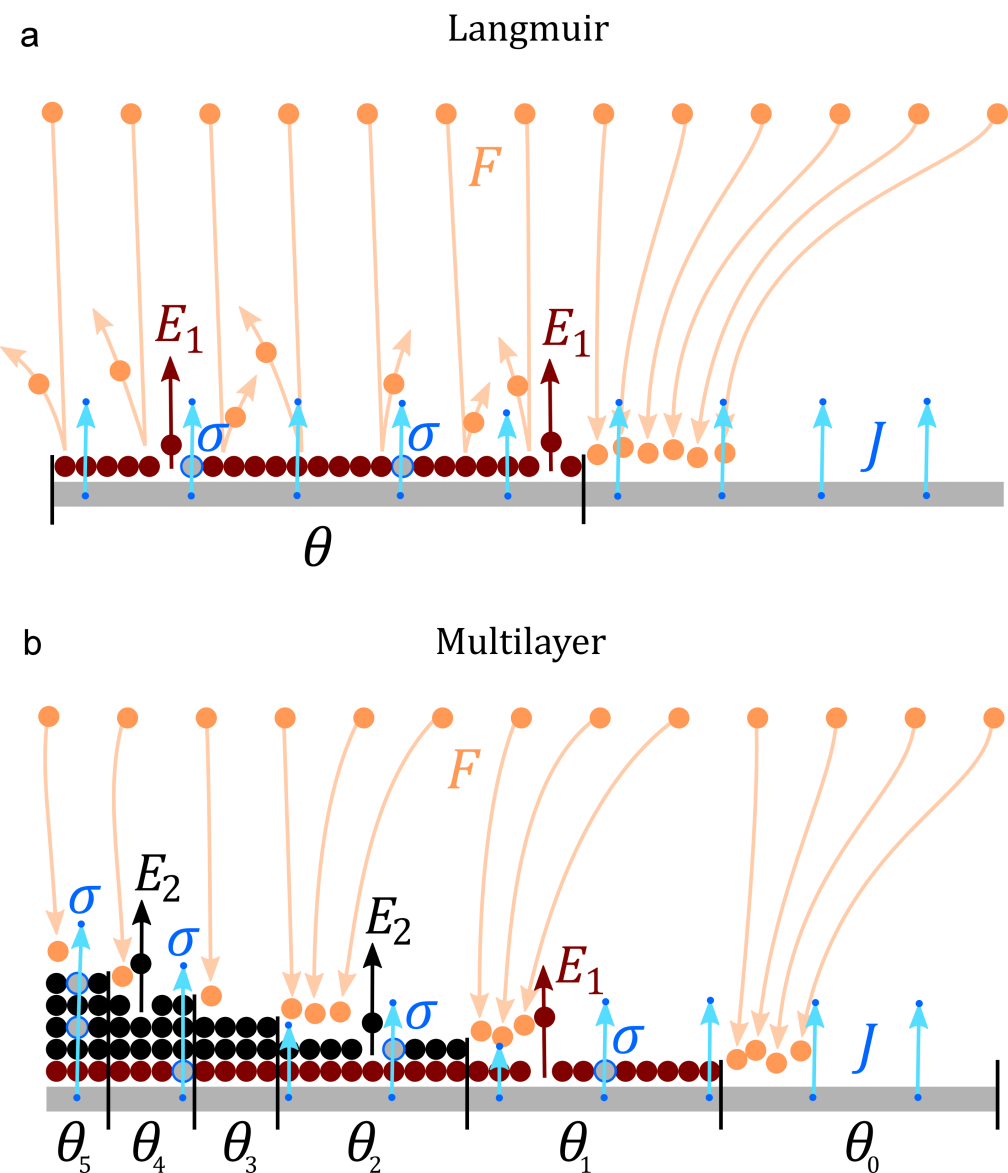
Schematic representation of the FEBID (a) Langmuir and (b) ML model. No diffusion is considered. Incoming precursor molecules with flux *F* are represented by orange spheres and arrows. Molecules adsorbed with energy *E*_1_ (for Langmuir and first monolayer in ML model) are represented with red spheres, and upper monolayers (ML model) adsorbed with energy *E*_2_ with black spheres. Electrons responsible for dissociation (here only represented as secondary electrons emitted from the substrate), with current density *J* and dissociation probability σ, are represented by blue dots and arrows. Molecules dissociated by electrons are shown as grey spheres. Only one monolayer is permitted in the Langmuir model, with fractional coverage θ. Multiple layers are possible in the ML model, where the fraction of empty sites is θ_0_, and fraction of occupied sites with one, two, three, etc. adsorbed monolayers, is θ_1_, θ_2_, θ_3_, etc._._

Both the Langmuir FEBID model and the multilayer (ML) FEBID model developed in this article are schematically compared in Figure 1. For Langmuir adsorption, the differential equation describing the time evolution for fractional molecule coverage θ = *N*/*N*_0_ is given by [25]:

[1]
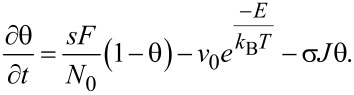


The first, second and third terms on the right hand side of Equation 1 refer to (Langmuir) adsorption, thermal desorption and electron dissociation, respectively, where *F* (molec/m^2^s) is the precursor flux, *s* the surface sticking coefficient, *N*_0_ (molec/m^2^s) the density of available sites, ν_0_ (1/s) is the thermal desorption attempt frequency, *E* (J) is desorption energy, *T* (K) is the temperature, *k*_B_ is Boltzmann’s constant, σ (m^2^) is the molecule dissociation cross section and *J* (1/m^2^s) is the electron flux density. No diffusion is considered, which is an approximation strictly valid under either no adsorbate concentration gradient conditions (which is only the case for negligible molecule depletion by electrons) or when the diffusive constant is very small [25,41]. The introduction of an additional diffusion term, proportional to the Laplacian of θ, would substantially complicate the analysis, requiring in general numerical methods to solve this and following equations both in time and space, which is beyond the scope of this work. More details about the validity of this approximation, in the context of the frequency analysis performed here, are given in Supporting Information File 1.

The ML model developed in this article follows the approach developed by Kusunoki [42], with time evolution for fractional coverage for empty sites θ_0_ and occupied sites with *i* monolayers θ*_i_* given by:

[2]
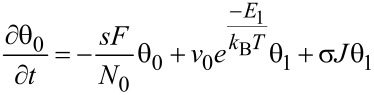


[3]
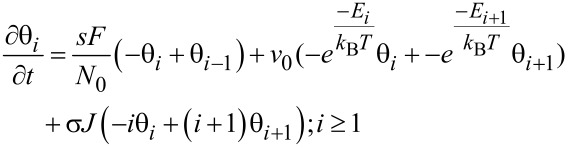


With *E_i_* = *E*_1_ for *i* = 1 and *E_i_* = *E*_2_ for *i* ≥ 2.

As before, the three right terms in Equation 2 and Equation 3 describe adsorption, desorption and dissociation effects, respectively, with prefactors as in Equation 1. However, now the fractional coverage of an area with *i* monolayers depends not only on that coverage, but also on areas covered by one more, or less, monolayers [42]. For instance, in Equation 2, the fraction of empty sites θ_0_ decreases over time due to incoming gas flux in a proportional way to θ_0_ (first term), and increases due to molecules being desorbed or dissociated, from areas occupied by one monolayer (second and third term, respectively). Analogously, in Equation 3, the time evolution of the fraction of occupied sites with *i* monolayers, θ*_i_*, is described by positive and negative terms involving θ*_i_*, θ*_i_*_+1_, and θ*_i−_*_1_. The model assumes that electrons will travel through the whole monolayer stack, with the probability of dissociating a molecule remaining unchanged along its path (see Figure 1b). The dissociation term σ*J*θ*_i_* is thus weighted by *i*, the number of monolayers present.

Importantly, the adsorption (first term) in Equation 3 is not self-limiting and allows for ML coverage. In addition, the second term includes Arrhenius factors with two desorption energies (*E*_1_ and *E*_2_), making it possible to describe two types of desorption processes. *E*_1_ is the interaction energy of the first monolayer with the substrate, whereas *E*_2_ is the desorption energy for all subsequent monolayers, representing the interaction between molecules adsorbed on top of each other. This approach is the same followed by the Brunauer, Emmet, Teller (BET) adsorption model [43], with a different desorption energy value for the first monolayer than for the rest, and *E*_2_ usually taken as the vaporization enthalpy. *E*_2_ is therefore the standard desorption energy employed in FEBID for physisorption [1]. The ML model presented here assumes several simplifications. First, chemisorption processes considered are spontaneous; energy barriers for activated chemisorption, which can be modelled via the inclusion of Arrhenius terms in the sticking coefficient [29], are not included. Second, the detailed adsorption state, coverage and order, as well as the electron irradiation, may significantly alter the values for attempt frequency, adsorption energy, and dissociation cross section, as well as the order of desorption [44–48]. These factors are not considered here but could be incorporated if necessary. In spite of its simplicity, the model is able to describe a rich phenomenology which goes beyond the standard Langmuir model usually considered for FEBID, enabling the study of multilayer systems. Moreover, it can describe processes involving both chemi- and physi-adsorption when *E*_1_ ≠ *E*_2_. This is essential when working on surfaces which may be chemically activated by electron irradiation [48]. Chemisorbed adsorbates are common, for instance, when using FEBID precursors leading to highly metallic deposits, such as Co_2_(CO)_8_, Co(CO)_3_NO and Fe(CO)_5_, where autocatalytic effects have been reported [15–16,31–32], as well as when mixing precursors with reactive gases in order to achieve high-purity deposits [10–14]. This model describes multilayer to monolayer transitions on activated deposit surfaces, therefore opening a new route to interpret this type of FEBID process.

### Representative cases for the multilayer model

We have investigated the steady state solution of the ML model and compare it with the Langmuir case. In line with previous works [3,19], it is convenient to define the following characteristic frequencies, which rule the behaviour of the system.

Here we define *v*_GAS_, the frequency for gas adsorption:

[4]
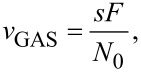


*v*_1_ as the frequency of gas desorption for the first monolayer (in contact with the substrate):

[5]
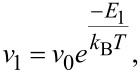


*v*_2_ as the frequency of gas desorption for upper monolayers (*i* ≥ 2):

[6]
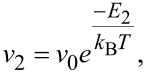


And *v*_e_ as the frequency of dissociation of adsorbed precursor

[7]
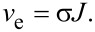


Table 1 shows examples of typical values for *v*_2_, *v*_e_ and *v*_GAS_, calculated for different standard experimental conditions, and using values extracted from literature. A wide range of frequency values can be accessed experimentally, with frequencies extending over several orders of magnitude. We include in Supporting Information File 1 details about how these have been calculated, as well as a spreadsheet as Supporting Information File 2 where the frequencies and their relative ratios are automatically calculated when introducing experimental parameters. This tool can be used in combination with the FEBID frequency maps explained in the next section.

**Table 1:** Examples of ν_2_, ν_e_ and ν_GAS_ values for standard experimental FEBID conditions. σ = 5·10^−21^ m^2^ for Co_2_(CO)_8_ is used from [19]. *F* is calculated for a Helios FEI dual beam system with a Pfeiffer TMH 262 turbo-molecular pump. Typical vaporization enthalpy values for FEBID precursors, associated to *E*_2_, are taken from [1]. Chemisorption energies *E*_1_ are significantly greater than *E*_2_ [1], leading to *v*_2_ << *v*_1_. See the spreadsheet in Supporting Information File 2 for more details.

ν_2_ [1/s]		Temperature [K]			ν_e_ [1/s]		Eff. spot diameter [nm]			ν_GAS_ [1/s]		GIS diameter [µm]		

		70	300	500			5	30	300			300	600	1200

Enthalpy [kJ/mol]	80	2·10^−47^	1·10^−1^	4·10^4^	Current [A]	10^−12^	4·10^3^	1·10^2^	1·10^0^	Growth *P* [mbar]	10^−7^	2·10^3^	6·10^2^	1·10^2^
	50	5·10^−25^	2·10^4^	6·10^7^		10^−9^	4·10^6^	1·10^5^	1·10^3^		10^−5^	2·10^5^	6·10^4^	1·10^4^
	30	4·10^−10^	6·10^7^	7·10^9^		10^−6^	4·10^9^	1·10^8^	1·10^6^		10^−3^	2·10^7^	6·10^6^	1·10^6^

Taking these frequencies into account, the steady state solution in the Langmuir case is given by:

[8]
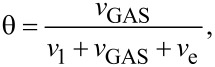


whereas for the ML model it takes the form:

[9]
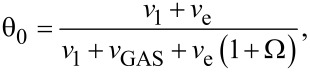


[10]
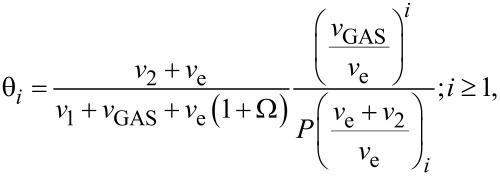


Where *P*()*_i_* is the Pochammer symbol and


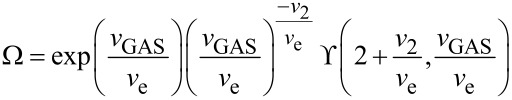


where 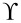 is the lower incomplete gamma function. As required, the ML model converges into the Langmuir model when *v*_2_ → ∞, corresponding to an infinitely fast desorption of all upper monolayers. See Supporting Information File 1 for more details.

**Figure 2:**
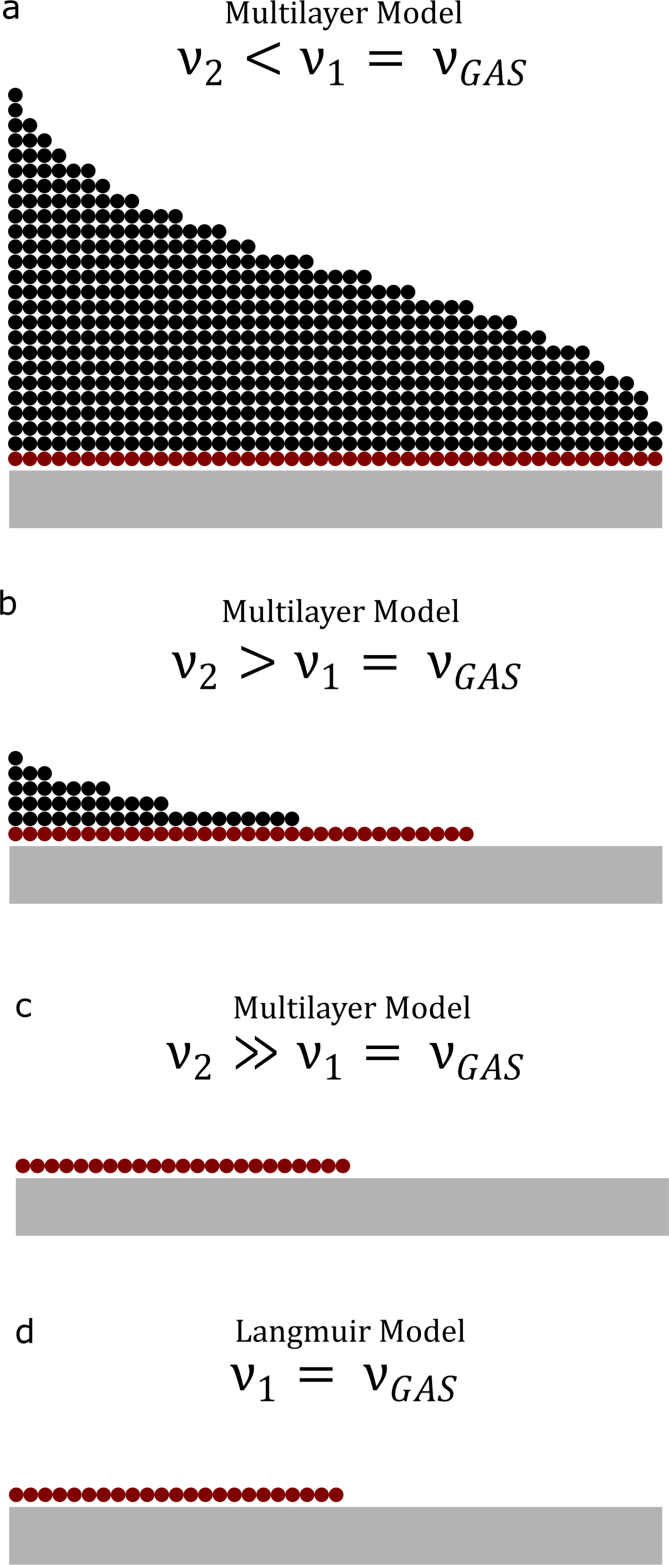
Three representative regimes described by the ML model (a, b, c), compared with the Langmuir model (d), under same conditions. The system was solved using *v*_1_ = *v*_GAS_ = 1, *v*_e_ = 0.04. Solutions are represented on a test surface with 45 adsorption sites.

In order to exemplify what type of steady state regimes may be reached in the ML model, Figure 2 shows fractional values for empty sites: first monolayer (red) and upper monolayers (black), for three representative cases, the result of solving Equation 9 and Equation 10 (Figure 2a–c) under varying *v*_2_. Steady states are calculated for *v*_2_ = *v*_GAS_, with a small dissociation frequency, *v*_e_ = 0.04. These cases are compared with the steady state reached solving the Langmuir model (Equation 8) for the same conditions, where a coverage of θ ≈ 0.5 is obtained (Figure 2d). For the case of strong physisorption (Figure 2a), characterized by a small ν_2_ value, the equilibrium state consists of a saturated first monolayer and a large number of layers on top. This is consequence of a slow desorption rate for physisorbed layers in comparison with the rate of molecule arrival.

On the contrary, for large ν_2_ values (Figure 2b), desorption of physisorbed molecules becomes faster than both molecule arrival (ν_GAS_) and desorption of the chemisorbed first monolayer (ν_1_). There exists, however, ML accumulation at the steady state, with the number of empty sites θ_0_ being lower than in the Langmuir limit (Figure 2d). This is due to two factors: First, all precursor arriving molecules contribute to an increase of coverage (Figure 1b) compared to the Langmuir case in which only those falling onto empty sites do (Figure 1a). Second, when a ML is formed, desorption from lower layers is inhibited, leading to an effective increase in the time required to desorb buried molecules. In the limiting case of *v*_2_ → ∞ (Figure 2c), physisorbed molecules are instantly desorbed, with the system converging to Langmuir coverage. These examples show the type of phenomenology described by the ML model. A fuller picture of possible FEBID regimes is presented in the next section.

### Langmuir and multilayer FEBID frequency maps

**Figure 3:**
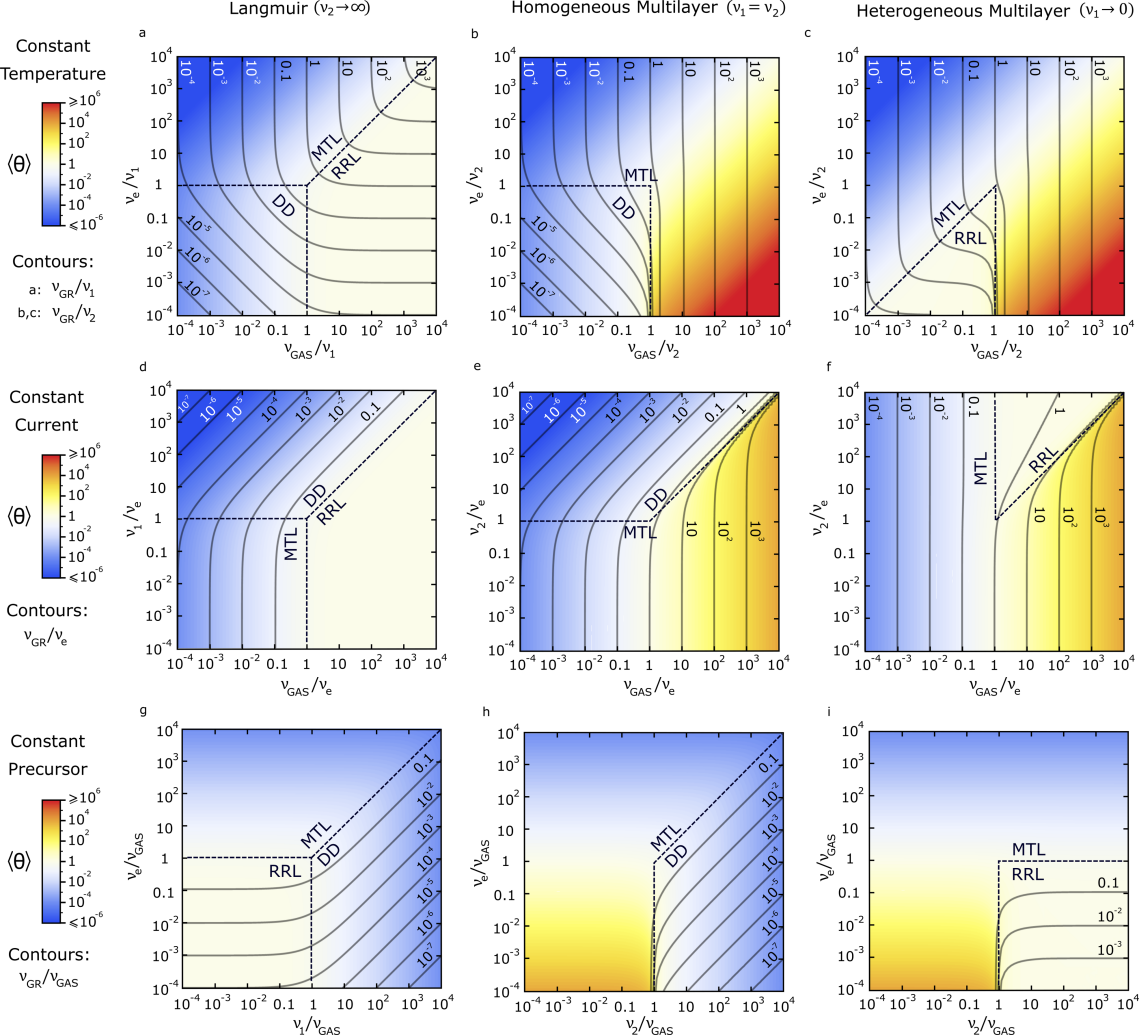
FEBID maps representing growth rate (contours) and adsorbate concentration (colours) for steady state conditions, as a function of the characteristic frequencies ruling a FEBID process. Different growth regimes are indicated for each region. Maps for constant temperature (a–c), current (d–f) and precursor flux (g–i) conditions are given. The Langmuir model (a, d, g), and the ML model with homogeneous adsorption (b, e, h) and heterogeneous adsorption (c, f, i) are compared.

In this section, we present a set of FEBID frequency maps, where growth rate and adsorbate coverage is plotted as a function of the fundamental frequencies determining the steady state of the system. These 2D maps describe, in a compact way, the general behaviour described by the Langmuir and ML models for a wide range of conditions (Figure 3), and can be used to design and understand FEBID experiments.

In order to construct such FEBID frequency maps, the average adsorbate coverage is given by

[11]
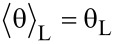


for the Langmuir case, and

[12]
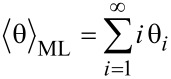


for the ML model.

Moreover, taking into account that the growth rate (m/s) is given by

[13]
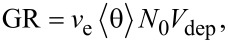


where *V*_dep_ (m^3^) is the deposited volume remaining after a molecule has been dissociated, we can define the growth rate frequency:

[14]
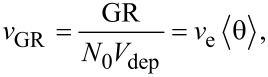


which represents the number of precursor monolayers that are being incorporated into the solid deposit each second. The addition of this frequency completes the frequency analysis presented above, with five characteristic frequencies {*v*_1_, *v*_2_, *v*_e_, *v*_GAS_, *v*_GR_}.

Figure 3 shows three types of maps, associated to three standard conditions: Constant temperature (Figure 3a–c), constant current (Figure 3d–f) and constant precursor flux (Figure 3g–i). Each case is normalized by the corresponding characteristic frequency (*v*_2_ or *v*_1_*, v*_e_ and *v*_GAS_, respectively). For each standard condition, the results are presented for the Langmuir model (left column), and for two relevant situations of the ML Model: homogeneous multilayer with *E*_1_ = *E*_2_ (middle column), and heterogeneous multilayer with *E*_1_ → ∞ (right column). The average coverage <θ> is represented with a colour scale and normalized growth rate frequency is represented as contour iso-lines. We discuss below some particular cases of areas and transitions between regimes observed in the maps. Cases not covered in the discussion can be understood with analogous arguments using the data in the maps.

To start, Figure 3a shows a paradigmatic example studied in FEBID: The transition from the mass-transport-limited (MTL) to the reaction-rate limited (RRL) regime at constant temperature [1–3] and Langmuir adsorption. The figure shows how RRL is found at high precursor fluxes, and is characterized by horizontal GR contours (i.e., independent of precursor flux) that are equally spaced vertically (GR proportional to current). In this case, <θ> tends to 1 (white colour). This contrasts with the case of high electron dissociation rates at high electron fluxes, with GR contours becoming vertical and equally spaced horizontally, and lower <θ> values (blue colour), all characteristics of the MTL. Additionally, in both the MTL and RRL regimes, the map shows how GR contours intersect the axes at the same value that they represent. This is a direct consequence of growth being respectively dominated by either dissociation (RRL) or molecule arrival (MTL) frequencies. The competition between frequencies is also evidenced by the transition between both regimes occurring at a diagonal line of slope = 1 (for *v*_e_/*v*_1_ > 1 and *v*_GAS_/*v*_1_ > 1). Additionally, the map shows a third regime when both dissociation and precursor adsorption frequencies fall below thermal desorption frequency values (*v*_e_/*v*_1_ < 1, *v*_GAS_/*v*_1_ < 1). In this desorption-dominated (DD) regime, <θ> is unsaturated (blue colour) and the gradient of the GR iso-lines is a diagonal with slope = 1, that is, the GR is equally linearly dependent on *v*_GAS_ and *v*_e_. A linear dependence on both gas flux and electron current has already been experimentally observed (see for instance [49]). In the DD regime, the horizontal (vertical) GR contour spacing is equivalent to the spacing in the MTL (RRL) regime, making it indistinguishable from the RRL and MTL when only one parameter is experimentally changed. This should be taken into account when analysing experimental data.

Figure 3b,c shows the same case discussed above, under constant temperature conditions, but for homogeneous MLs: ν_1_ = ν_2_ (Figure 3b) and heterogeneous MLs: ν_1_ → 0 (Figure 3c). There are key differences between the Langmuir and ML models: Firstly, when ML formation is allowed, the system becomes mostly MTL since the ability of the substrate to accept molecules is increased greatly. Secondly, for homogenous ML systems with only physisorption (Figure 3b), the RRL regime disappears, whereas for heterogeneous MLs with high chemisorption energies (Figure 3c), there is no DD regime. The lack of RRL for a homogeneous ML model is a consequence of the disappearance of any saturation mechanism for surface coverage, as occurs for Langmuir adsorption (see how the white colour in Figure 3a, corresponding to <θ> = 1, becomes yellow and red, i.e., <θ> > 1, in Figure 3b). On the other hand, the absence of a DD region for the heterogeneous ML model is due to the presence of a strongly chemisorbed first monolayer (<θ> = 1: white color), formed even for low gas fluxes, and insensitive to changes in temperature due to a high *E*_1_ value. Under these circumstances, the growth at low *v*_GAS_/*v*_2_ ratios is mostly RRL. Finally, an important difference between the models is related to GR values: Whereas for Langmuir systems, the spacing between nearby GR contours does not show large changes, in ML systems, very steep changes happen at the conditions where ML formation is fulfilled (see e.g. area around *v*_GAS_ / *v*_2_ = 1 in Figure 3b,c).

The differences discussed between the models for constant temperature conditions are equally reflected in the maps at constant current (Figure 3d vs Figure 3e,f) and constant precursor flux (Figure 3g vs Figure 3h,i) conditions. For instance, a constant white colour readily identifies the RRL regime, whereas a colour change from white to yellow/red indicate transitions from monolayer to ML coverage (at ν_GAS_ = ν_2_ > ν_e_), characterized by steep changes in GR.

### FEBID isotherms

**Figure 4:**
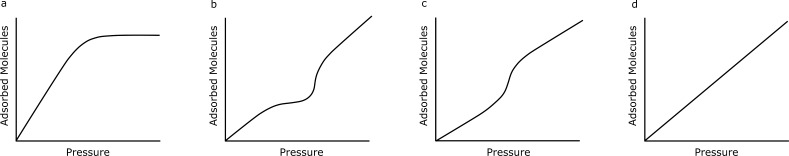
FEBID adsorption isotherms extracted from FEBID frequency maps shown in Figure 3d–f. (a) Type I (Langmuir), (b) Type II (BET), (c) Type III, and (d) Linear (MTL regime).

The maps in Figure 3d–f can be employed to extract FEBID isotherms (surface coverage vs pressure at constant temperature) for different models and regimes. These are obtained by evaluating the dependence of <θ> with *v*_GAS_/*v*_e_ for constant *v*_2_/*v*_e_ values, that is, via the colour evolution along horizontal lines. Changes in the slope of the isotherms are easy to observe via horizontal changes in the separation between GR contours. Using the classic classification of adsorption isotherms [50], the types of FEBID isotherms extracted from the maps are shown in Figure 4. In the Langmuir case (Figure 3d), all isotherms are of type I (Figure 4a). Any horizontal line in the diagram transitioning from the DD (or MTL) to the RRL regime goes from blue to white, where it saturates. On the contrary, when MLs are considered (Figure 3e,f), different types of isotherms are observed: Linear isotherms (Figure 4d) are found in both cases for *v*_2_/*v*_e_ < 1, as expected for the MTL regime. This linear isotherm is not one of the five standard types [50], since FEBID curves discussed here include the electron dissociative term, in contrast with standard adsorption isotherms. A different scenario is observed for *v*_2_/*v*_e_ > 1 in Figure 3e, where concave type III-like isotherms (Figure 4c) are found, due to the system transitioning from the DD to the MTL regime. These isotherms are a consequence of the ML formation before the saturation of the first monolayer, for homogeneous ML adsorption conditions. Finally, concave–convex type II-like BET isotherms (Figure 4b), given by evolution from blue to temporarily saturated white, followed by unsaturated yellow, are observed for heterogeneous MLs with *v*_2_/*v*_e_ > 1 (Figure 2f). Here, the system transitions first from MTL to RRL, followed by a transition to the MTL regime again. The last transition to the MTL regime is triggered by the formation of MLs, with the first monolayer being already saturated due a large chemisorption energy value *E*_1_. The FEBID type II and III isotherms discussed here are analogous to the classic ones. However, whereas classic isotherms diverge quickly at high pressures [50], here the dependence with pressure is linear (when at the MTL). This is due to the inclusion of an electron dissociative term in the ML model that depends linearly with the number of monolayers adsorbed (Equation 3). Moreover, type IV and V multilayer isotherms [50], which are variants of type II and III, respectively, with <θ> becoming saturated at high pressures, are not found in these FEBID frequency maps, since we are not considering any ML saturation mechanism here. Saturation due to effects such as a fast decrease of adsorption energy with number of adsorbed layers or filling of porous media could be readily taken into account by truncating the corresponding sum series [43] in Equation 3.

### Analysis of experimental data using FEBID frequency maps

As a practical demonstration, we analyse an example selected from the literature within the framework of the FEBID frequency maps. This type of analysis can be performed only for previous publications where a comprehensive set of experimental conditions have been reported. This is not the case in most FEBID publications, as previously highlighted [2].

**Figure 5:**
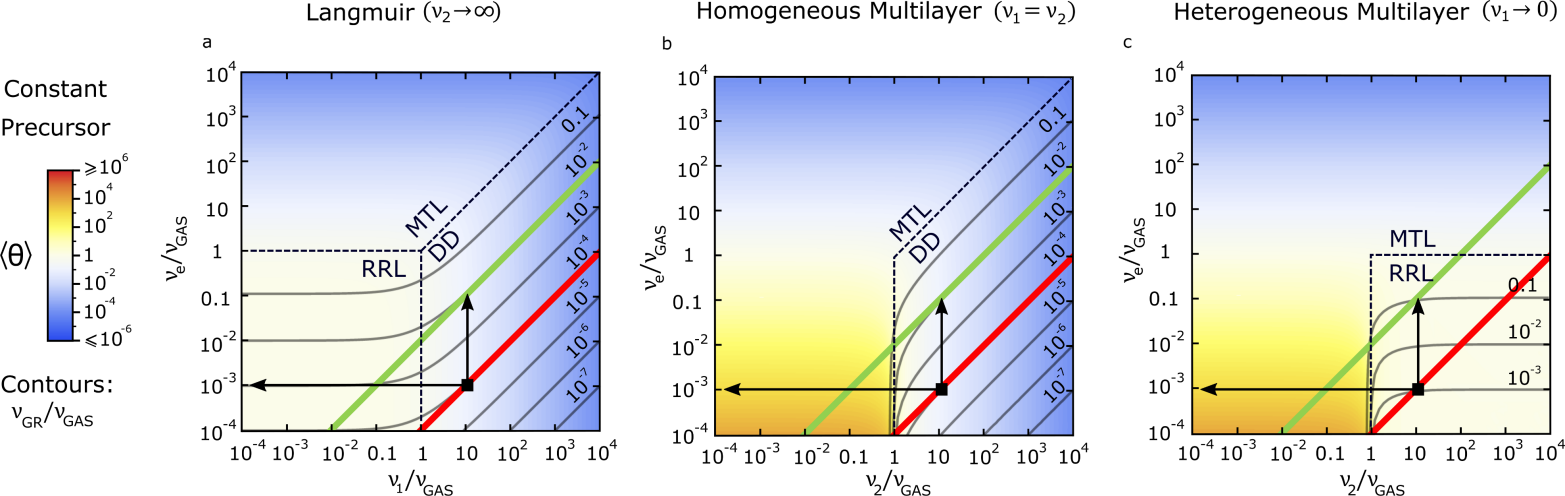
Analysis of [37] by Bresin et al. using FEBID frequency maps under constant precursor flux, that is, using Figure 3g–i and frequencies extracted from the experimental data. Three possible scenarios are considered: the Langmuir model (a), the ML model with homogeneous (b) and heterogeneous (c) adsorption. Red and green lines correspond to the estimated areas where experiments take place. These regions are estimated just as lines, and not as points, since the value for ν_GAS_ is not available. Red line: Room temperature, low current. Green line: Room temperature, high current. The low temperature, low current region takes place at much lower ν_2_ / ν_GAS_ values as those plotted in the map. Vertical arrows represent one of the possible experimental transitions in GR observed at room temperature, from low to high current. Horizontal arrows represent one of the possible GR transitions observed at low current, from room to low temperature.

In [37], Bresin et al. report cryogenic FEBID experiments using MeCpPtMe_3_, obtaining GRs as a function of temperature and electron current, for constant gas flux conditions (Figure 3g–i is thus employed for this analysis). The precursor condensation on the surface at cryogenic temperatures is exploited, which is a rather extreme case of ML formation. Taking *E*_2_ = 56 kJ/mol as the vaporization enthalpy for this precursor at high coverages [51], *v*_0_ = 10^13^ s^−1^ as the desorption attempt frequency [52], and *T* = 120–300 K as the temperature range investigated, *v*_2_ is estimated to change in a very wide range, from 10^−12^ s^−1^ to 10^3^ s^−1^ (Equation 6). From the range of electron fluxes reported (10^7^–10^9^ µC/m^2^s) and a dissociation cross section of σ = 10^−20^ m^2^ [51], *v*_e_ ranges approximately from 10^−1^ to 10 s^−1^ (Equation 7). *v*_GAS_ cannot be estimated from the available data. The relative magnitude estimated for *v*_e_ and *v*_2_ restrict the experiments to the following area (see Figure 5): from *v*_2_ ≈ 10^4^·*v*_e_ at room temperature and low current (red line) to *v*_2_ ≈ 10^2^·*v*_e_ at room temperature and high current (green line, parallel to the red one). The drop in *v*_2_ when moving to cryogenic temperatures is too large to be represented in the figure; low temperature experimental conditions would be therefore represented by a line parallel to the red one, but horizontally shifted to the left, reaching a range beyond the *x*-axis. The above discussion applies to the Langmuir case, with *v*_1_ replacing *v*_2_. To estimate which model is most appropriate to describe this experiment, we employ the GR changes reported: A two order of magnitude increase, from low to high current at room temperature, as well as a four order of magnitude increase, from room temperature to cryogenic temperatures, when working at low current, are observed. In all three models (see Figure 5a–c), a two order of magnitude increase in GR with increasing current is observed for a vertical transition from red to green lines (see vertical black arrows for one example). However, only the case of homogeneous physisorbed MLs (Figure 5b) is consistent with the four order of magnitude increase in GR when moving towards cryogenic temperatures (see horizontal black arrows for one example). A one order of magnitude increase is predicted by the Langmuir model (Figure 5a), and a three order of magnitude, at most, by the heterogeneous ML model (Figure 5c).

There exists an important degree of uncertainty in this analysis, since the frequency ranges considered are just an estimation. However, the formation of MLs under homogeneous adsorption at low temperatures is indeed plausible for these experiments. This discussion is an illustration of the usefulness of FEBID frequency maps for the analysis of experimental data, which could trigger the design of new experiments as well. For instance, in this particular case, additional measurements of GR as a function of precursor flux would make the distinction between the homogeneous and heterogeneous ML cases possible. In addition, GRs for electron current density changing over several orders of magnitude would be useful to accurately determine the transition from the DD to the MTL regime.

### Guiding future experiments

In order to gain full advantage of the tools presented in this work, Supporting Information File 2 includes a frequency calculator spreadsheet, where a FEBID user should insert the different parameters necessary to calculate FEBID fundamental frequencies. Among all these parameters, it includes those which can be easy to access experimentally (e.g., chamber pressure, beam energy, beam current, GIS diameter), and should be recorded during a standard FEBID experiment. In addition to these, another set of parameters included in the spreadsheet, such as molecule scattering cross section, sticking factor, desorption energy, can be either accessed from previous literature (see [1] for a large compilation) or extracted experimentally via dedicated experiments [52–53]. Supporting Information File 1 gives a more detailed discussion on how to use the spreadsheet and what parameters are required to obtain the different FEBID characteristic frequencies.

It should be noted, as discussed in the previous section, that there exists a significant uncertainty in the estimation of these frequencies, in the region of the map where an experiment takes place. This is as a consequence of the difficulty in obtaining some of these parameters, and the fact that they may have been measured at different conditions, casting doubts about their validity for a different experiment. To reduce this uncertainty, GR values obtained experimentally should be compared with those estimated with Equation 13 for a given point in the map, allowing assessment of the valitity of the estimations made. For systems at the MTL regime, the diffusion term may become relevant (see Supporting Information File 1), making the GR values obtained from the maps a lower bound of the actual rates that may be obtained experimentally. Moreover, of particular interest is the design of experiments which probe transitions between different regimes: In the crossover between regimes, characteristic frequencies balance, providing an opportunity to experimentally quantify fundamental parameters.

Finally, we propose the design and realization of experiments similar to the one discussed in the previous section, that is, where two parameters (e.g. electron current and gas flux) are independently changed (represented by horizontal and vertical lines in a FEBID map) over a wide range, leading to two different crossovers between regimes. By following this procedure, and measuring the number of orders of magnitude that each frequency needs to be varied for the two crossovers to occur, it should be possible to accurately find the working area of an experiment in its corresponding FEBID frequency map.

## Conclusion

In summary, we have extended the FEBID continuum model beyond Langmuir adsorption, allowing for adsorbate coverages above one monolayer. We have used the approach followed by the BET model, introducing two types of adsorption energies: one accounting for molecule–substrate interaction and a second for molecule–molecule interaction in upper monolayers. This generalizes the range of applicability of the FEBID continuum model by including processes involving multilayer formation, which are typical at low temperatures and for heterogeneous substrates. It also enables the modelling of FEBID processes occurring on activated deposit surfaces, where both chemisorption and physisorption processes are relevant, opening a new route to interpret results where high purity deposits have been reported. The approximations followed by the model, and ways to make it more complex, are briefly described.

We have determined the stationary state for the Langmuir and multilayer models under no diffusion, studying the possible regimes reached under a wide range of conditions. All phenomena observed, including the formation of saturated monolayers, appearance of multilayers, or the convergence from the multilayer model to the Langmuir model, can be understood in terms of the characteristic time scales of the system. These results are synthesized in a set of dimensionless FEBID frequency maps for Langmuir, and homogeneous and heterogeneous multilayer adsorption conditions. We have identified three fundamental FEBID regimes, corresponding to mass-transport limited, reaction-rate limited and desorption-dominated conditions. Moreover, we extract and classify the types of FEBID isotherms described by these models, finding four types of curves, which are analogous to those described by the adsorption isotherm theory, but include the electron dissociative term.

Finally, we propose FEBID frequency maps as a new tool to analyze experimental data in detail, and as an aid to identify possible steady states and transitions between growth regimes. This analysis emphasizes the need for investigating and reporting FEBID deposits as a function of multiple experimental conditions. A frequency calculator is included as Supporting Information File 2 to facilitate the usage of FEBID frequency maps in other works.

## Supporting Information

File 1 Additional information on the model.
Analytical solution of the steady state model and a guide for the calculation of characteristic frequencies.

File 2 FEBID frequency tool.
A spreadsheet for the quick calculation of characteristic frequencies.
