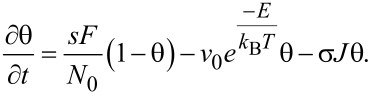# Correction: Modelling focused electron beam induced deposition beyond Langmuir adsorption

**DOI:** 10.3762/bjnano.8.259

**Published:** 2017-12-05

**Authors:** Dédalo Sanz-Hernández, Amalio Fernández-Pacheco

**Affiliations:** 1 Cavendish Laboratory, University of Cambridge, JJ Thomson Cambridge, CB3 0HE, United Kingdom

**Keywords:** adsorption isotherm theory, BET model, continuum model, focused electron beam induced deposition, 3D nanoprinting, Langmuir model

The originally published Equation 1 contains a mistake. The theta factor in the second right term is missing. The correct Equation 1 is

[1]