# Supplementary material for: Modelling focused electron beam induced deposition beyond Langmuir adsorption
Source: Beilstein J Nanotechnol. 2017 Oct 13;8:2151–61. doi: 10.3762/bjnano.8.214 (PMC5647696; doi:10.3762/bjnano.8.214)
Supplement: File 1 — Additional information on the model. Analytical solution of the steady state model and a guide for the calculation of characteristic frequencies. [file Beilstein_J_Nanotechnol-08-2151-s001.pdf]

Supporting Information

for

Modelling focused electron beam induced deposition beyond Langmuir adsorption

Dédalo Sanz-Hernández[1] and Amalio Fernández-Pacheco[1*]

Address: [1]Cavendish Laboratory, University of Cambridge, JJ Thomson Cambridge, CB3 0HE, United Kingdom

Email: Amalio Fernández-Pacheco - af457@cam.ac.uk

* Corresponding author

Additional Information on the Model

In order to comply with EPSRC policy, the metadata associated to this publication can be found here:
https://doi.org/10.17863/CAM.11214

Part 1: Analytical solution to the Multilayer model

For the Multilayer (ML) model, the following equations applies, where θ_i is defined as the fractional coverage of sites with n monolayers:

$$\frac{\partial\theta_0}{\partial t} = \frac{sF}{N_0}\theta_0 + \sigma J\theta_1 + \nu_0 e^{\frac{-E_1}{k_B T}}\theta_1 \quad (1)$$

$$\frac{\partial\theta_i}{\partial t} = \frac{sF}{N_0}(\theta_{i-1} - \theta_i) + \sigma J\big((i+1)\theta_{i+1} - n\theta_i\big) - \nu_0 e^{\frac{-E_i}{k_B T}}\theta_i + \nu_0 e^{\frac{-E_{i+1}}{k_B T}}\theta_{i+1} \ ; \ i \geq 1 \quad (2)$$

with $E_i = E_2$ for $i \geq 2$.

Both equations can be solved analytically, being convenient to define the following characteristic frequencies of the system before presenting the solutions:

- $\nu_{GAS} = \frac{sF}{N_0}$, the frequency of precursor gas addition
- $\nu_e = \sigma J$, the frequency of precursor gas dissociation
- $\nu_1 = \nu_0 e^{\frac{-E_1}{k_B T}}$, the frequency of precursor gas desorption from chemisorbed sites
- $\nu_2 = \nu_0 e^{\frac{-E_2}{k_B T}}$, the frequency of precursor gas desorption from physisorbed sites

To solve the system, we first find, from the steady state condition, the population ratios:

$$\frac{\theta_1}{\theta_0} = \frac{\nu_{GAS}}{\nu_1 + \nu_e} \quad (3)$$

$$\frac{\theta_i}{\theta_{i-1}} = \frac{\nu_{GAS}}{\nu_2 + i\nu_e} \ ; \ i > 1 \quad (4)$$

From these ratios, the population θ_i is defined as:

$$\theta_i = \theta_0 \prod_{p=1}^{i} \frac{\theta_p}{\theta_{p-1}} \quad (5)$$

that for $i > 1$ is

$$\theta_{i>1} = \theta_0 \frac{\nu_{GAS}}{\nu_1 + \nu_e} \prod_{p=2}^{i} \frac{\nu_{GAS}}{\nu_2 + p\nu_e}$$

which was simplified using Wolfram Mathematica to

$$\theta_i = \theta_0 \frac{\nu_2 + \nu_e}{\nu_1 + \nu_e} \frac{\left(\frac{\nu_{GAS}}{\nu_e}\right)^i}{\left(1 + \frac{\nu_2}{\nu_e}\right)_i} ; \ i > 1 \quad (6)$$

$(\,)_i$ being the Pochhammer symbol.

A value for θ_0 can then be solved by applying the constrain

$$\sum_{i=0}^{\infty} \theta_i = 1 \tag{7}$$

which means that

$$\theta_0 + \theta_0 \frac{\nu_{GAS}}{\nu_1+\nu_e} + \theta_0 \sum_{i=2}^{\infty} \frac{\nu_2+\nu_e}{\nu_1+\nu_e} \frac{\left(\frac{\nu_{GAS}}{\nu_e}\right)^i}{\left(1+\frac{\nu_2}{\nu_e}\right)_i} = 1 \tag{8}$$

which was solved using Wolfram Mathematica to

$$\theta_0 = \frac{\nu_1+\nu_e}{\nu_1+\nu_{GAS}+\nu_e(1+\Omega)} \tag{9}$$

with $\Omega = e^{\frac{\nu_{GAS}}{\nu_e}} \left(\frac{\nu_{GAS}}{\nu_e}\right)^{\frac{-\nu_2}{\nu_e}} \Upsilon\left(2+\frac{\nu_2}{\nu_e}, \frac{\nu_{GAS}}{\nu_e}\right)$, Υ being the lower incomplete gamma function.

Combining (S6) with (9) and (S3) we readily obtain

$$\theta_i = \frac{\nu_2+\nu_e}{\nu_1+\nu_{GAS}+\nu_e(1+\Omega)} \frac{\left(\frac{\nu_{GAS}}{\nu_e}\right)^i}{P\left(\frac{\nu_e+\nu_2}{\nu_e}\right)_i} \; ; \; i \geq 1 \tag{10}$$

Part 2: Convergence to the Langmuir limit

As expected, convergence of the ML model into the Langmuir model happens when $\nu_2 \to \infty$ (infinitely fast desorption of physisorbed molecules).

Using

$$\lim_{\nu_2\to\infty} \Omega = 0 \tag{11}$$

$$\left(\frac{\nu_e+\nu_2}{\nu_e}\right)_1 = \frac{\nu_e+\nu_2}{\nu_e} \tag{12}$$

$$\lim_{\nu_2\to\infty} \left(\frac{\nu_e+\nu_2}{\nu_e}\right)_{n>0} = \infty \tag{13}$$

leads to:

$$\lim_{\nu_2\to\infty} \theta_1 = \frac{\nu_{GAS}}{\nu_1+\nu_{GAS}+\nu_e} = \theta_L \tag{14}$$

$$\lim_{\nu_2\to\infty} \theta_0 = \frac{\nu_1+\nu_e}{\nu_1+\nu_{GAS}+\nu_e} = 1-\theta_L \tag{15}$$

$$\lim_{\nu_2\to\infty} \theta_{n>1} = 0 \tag{16}$$

Part 3: Notes on diffusion

The framework developed in this work assumes no diffusion. Diffusion is however not negligible in many FEBID cases, in particular in the presence of strong electron-induced molecule depletion.

The continuum model diffusive term has the form $D\nabla^2\theta$, where D[m²/s] is the diffusion coefficient, ∇^2 the Laplace operator and θ the surface concentration of precursor. The diffusion coefficient is given by $D = D_0 e^{-E_{diff}/k_B T}$, with E_{diff} [J] the energy barrier to diffusion and D_0 [m²/s] the diffusion constant in the limit of inexistent energy barriers [1]. The units of $\nabla^2\theta$ are [m^{-2}].

Following the same discussion on characteristic frequencies as in the main manuscript, we can define a diffusion frequency $\nu_{diff} = D\nabla^2\theta$ [s^{-1}], describing how often precursor molecules arrive to, or leave, a given site due to diffusion. This term will be, generally, space dependent. If ν_{diff} is the smallest of the set $\{\nu_1, \nu_2, \nu_e, \nu_{GAS}, \nu_{diff}\}$ by at least an order of magnitude then diffusion can be neglected.

In most FEBID experiments, the main source of $\nabla^2\theta$ is the profile of electron irradiation, which can cause a reduction of θ at the region of higher electron irradiation. We can therefore also assess the relevance of diffusion in a system by evaluating the level of depletion caused by the electron beam:

$$Electron\ Induced\ Depletion = \frac{\Delta\langle\theta\rangle}{\langle\theta\rangle(\nu_e=0)} \qquad (17)$$

with $\Delta\langle\theta\rangle = \langle\theta\rangle(\nu_e = 0) - \langle\theta\rangle(\nu_e)$ being the difference between average coverage under no electron irradiation $\langle\theta\rangle(\nu_e = 0)$, and coverage under irradiation $\langle\theta\rangle(\nu_e)$. The expression for the coverage is given by eqs. 11 and 12 in the main manuscript and represented in the FEBID frequency maps. The regimes in which no depletion will occur are directly readable from the constant precursor maps of Fig.3 in the main manuscript, included below for convenience.

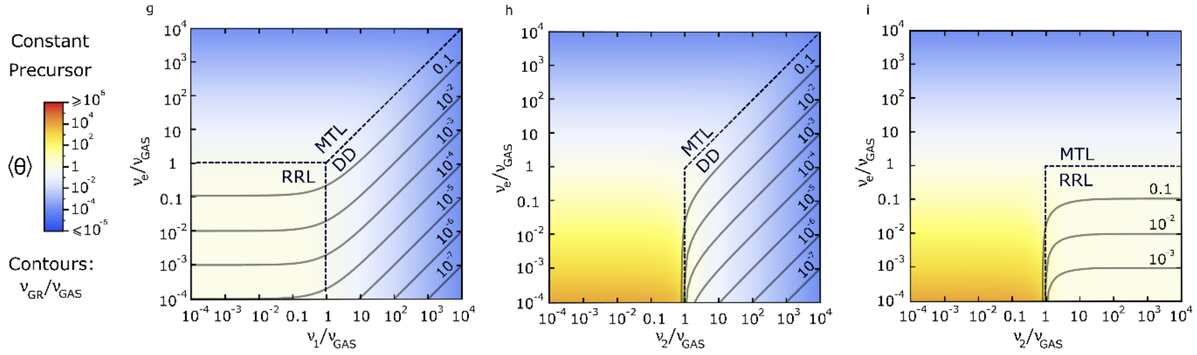

Fig. 3g, h, i of the main manuscript, included here to help in this discussion.

The bottom of this maps (horizontal axis) corresponds to very low electron irradiation, with the color at this area as a good approximation to $\langle\theta\rangle(\nu_e = 0)$. In order to see if electron-induced depletion occurs in a regime, we just need to see if $\langle\theta\rangle$ drops when moving vertically upwards in the map. In line with previous discussion in the literature [2], depletion only happens in the MTL regime. It is therefore only necessary to consider diffusion in this case. This does not imply, however, that diffusion will necessarily play a role in every MTL system, since even in the presence of electron-induced depletion, the diffusion coefficient D may still be very small. An illustrative numerical study of this case is given by [1], where the authors illustrate how, in a transition from the MTL to the DD regime as a function of temperature, the contribution of diffusion is first negligible, then becomes significant due to the activation of diffusion at $k_B T \approx E_{diff}$, and disappears at higher temperatures when desorption becomes dominant. For an extended analysis of systems in the MTL regime with diffusion, we refer

the readers to [3,4], where the authors describe the different deposit shapes resulting from diffusion and the effect of using different electron beam profiles amongst other important considerations.

Part 4: FEBID frequency calculator

The FEBID frequency calculator is a Microsoft Excel document, included as well as Supporting Information, which can be used to estimate the relevant frequencies from experimentally available parameters. The frequency ratios described in the main manuscript are calculated, which makes it possible to locate a given experiment within the FEBID maps shown in Fig. 3. of the main manuscript.

On the left side of the calculator, the experimental data is entered, which results in an estimation of the corresponding frequencies. At the top, an evaluation of the ratios between them. For further reference, a set of tables for typical FEBID cases is generated on the right. The six reference values used to generate each table (e.g. temperatures and enthalpies in the case of ν_2) can be changed by the user.

Parameter	Unit
Precursor used	-
Chamber Residual Pressure	Pa
Chamber Growth Pressure	Pa
Chamber Pump speed	l/s
Chamber Temperature	K
Substrate material	-
Substrate temperature	K
GIS diameter	m
GIS to substrate distance	m
GIS to substrate angle	degrees
Beam energy	keV
Beam current	A
Beam width	m

Table S1: Experimental checklist for every experiment which is to be analyzed using characteristic frequencies.

As a general rule, if an experimental result is to be analyzed using this method, every variable outlined in Table S1 should have been recorded.

The calculation of ν_1 and ν_2 is based on the following equations:

$$\nu_1 = \nu_0 e^{\frac{-E_1}{k_B T}} \qquad (17)$$

$$\nu_2 = \nu_0 e^{\frac{-E_2}{k_B T}} \qquad (18)$$

where ν_0 is typically 10^{13} s^{-1} [ref. 2], and the values for E_1 and E_2 are either obtained directly from the literature or calculated from the condensation enthalpy as:

$$E = \frac{H_{Cond}[\text{kJ/mole}]}{N_A}\frac{1000[\text{kJ}]}{1[\text{J}]} \qquad (19)$$

where N_A is Avogadro's number.

The calculation of ν_{GAS} is more involved. First, the density of sites (N_0) is estimated under the 'Precursor Properties' section assuming square-packed molecules on the substrate. Then the molecule flux from the GIS is estimated assuming that most of the gas being pumped out during growth is coming from the GIS. Therefore, we assume that the volume adsorbed onto the substrate is negligible when compared to the volume of gas ejected by the GIS. Applying conservation of mass, the number of molecules exiting the GIS is every second (M_{GIS}) is the same as the extra number of molecules being extracted by the pump, which is given by

$$S_{PUMP}[s^{-1}] = \frac{\Delta P[\text{Pa}]}{K_B\left[\frac{J}{\text{K}}\right]T[\text{K}]}Q_{PUMP}\left[\frac{\text{litres}}{s}\right]\frac{1[\text{m}^3]}{1000[\text{litres}]} \qquad (20)$$

where K_B is Boltzmann's constant, Q_{PUMP} is the volumetric flow rate of the pump at the molecular mass of the precursor and ΔP is the rise in pressure during growth.

The flux exiting the GIS is

$$F_{GIS}[\text{s}^{-1}\text{m}^{-2}] = \frac{S_{PUMP}[s^{-1}]}{A_{GIS}[\text{m}^2]} \qquad (21)$$

where A_{GIS} is the cross sectional area of the GIS. To estimate the flux at the substrate, a geometrical correction g is applied [5], leading to

$$F = gF_{GIS} \qquad (22)$$

Under these assumptions, we can calculate the frequency for gas adsorption:

$$\nu_{GAS} = \frac{sF}{N_0} \qquad (23)$$

where s is the fraction of molecules which stick to the substrate upon arrival.

To estimate the electron dissociation frequency, the density of electrons at the surface is first estimated using electron beam current, expected secondary electron yield γ, and an effective beam diameter I taken as the diameter of the electron-substrate interaction volume.

The current density J at the substrate surface is then

$$J[\text{s}^{-1}\text{m}^{-1}] = \gamma\frac{I[\text{C/s}]}{\pi\left({}^{d[\text{m}]}/_{2}\right)^2 e[\text{C}]} \qquad (24)$$

where e is the electron charge.

This allows calculating ν_e as

$$\nu_e = \sigma J \qquad (25)$$

with the dissociation cross section σ value, generally extracted from the literature.

References

(1) Cullen, J.; Lobo, C. J.; Ford, M. J.; Toth, M. *ACS Appl. Mater. Interfaces* **2015**, *7* (38), 21408–21415.

(2) Cullen, J.; Bahm, A.; Lobo, C. J.; Ford, M. J.; Toth, M. *J. Phys. Chem. C* **2015**, *119* (28), 15948–15953.

(3) Szkudlarek, A.; Szmyt, W.; Kapusta, C.; Utke, I. *Appl. Phys. A* **2014**, *117* (4), 1715–1726.

(4) Toth, M.; Lobo, C.; Friedli, V.; Szkudlarek, A.; Utke, I. *Beilstein J. Nanotechnol.* **2015**, *6*, 1518–1540.

(5) Friedli, V.; Utke, I. *J. Phys. D. Appl. Phys.* **2009**, *42* (12), 125305.
